# Deep CO_2_ release and the carbon budget of the central Apennines modulated by geodynamics

**DOI:** 10.1038/s41561-024-01396-3

**Published:** 2024-04-19

**Authors:** Erica Erlanger, Aaron Bufe, Guillaume Paris, Ilenia D’Angeli, Luca Pisani, Preston Cosslett Kemeny, Jessica Stammeier, Negar Haghipour, Niels Hovius

**Affiliations:** 1 GFZ German Research Centre for Geosciences, Potsdam, Germany; 2 Centre de Recherches Pétrographiques et Géochimiques, CRPG UMR 7358, Université de Lorraine–CNRS, Nancy, France; 3 Department of Earth and Environmental Sciences, Ludwig–Maximilians–Universität München, Munich, Germany; 4 Department of Geosciences, Università di Padova, Padua, Italy; 5 Department of Biological, Geological, and Environmental Sciences, Università di Bologna, Bologna, Italy; 6 Biblioteca Franco Anelli, Società Speleologica Italiana, Bologna, Italy; 7 Department of the Geophysical Sciences, The University of Chicago, Chicago, IL USA; 8 Laboratory of Ion Beam Physics, ETH Zürich, Zurich, Switzerland; 9 Geological Institute, ETH Zürich, Zurich, Switzerland; 10 Institute of Geosciences, University of Potsdam, Potsdam, Germany

**Keywords:** Hydrology, Geodynamics, Tectonics, Climate sciences

## Abstract

Recent studies increasingly recognize the importance of critical-zone weathering during mountain building for long-term CO_2_ drawdown and release. However, the focus on near-surface weathering reactions commonly does not account for CO_2_ emissions from the crust, which could outstrip CO_2_ drawdown where carbonates melt and decarbonize during subduction and metamorphism. We analyse water chemistry from streams in Italy’s central Apennines that cross a gradient in heat flow and crustal thickness with relatively constant climatic conditions. We quantify the balance of inorganic carbon fluxes from near-surface weathering processes, metamorphism and the melting of carbonates. We find that, at the regional scale, carbon emissions from crustal sources outpace near-surface fluxes by two orders of magnitude above a tear in the subducting slab characterized by heat flow greater than 150 mW m^–2^ and crustal thickness of less than 25 km. By contrast, weathering processes dominate the carbon budget where crustal thickness exceeds 40 km and heat flow is lower than 30 mW m^–2^. The observed variation in metamorphic fluxes is one to two orders of magnitude larger than that of weathering fluxes. We therefore suggest that geodynamic modulations of metamorphic melting and decarbonation reactions are an efficient process by which tectonics can regulate the inorganic carbon cycle.

## Abstract (web summary)

The regional geodynamic gradient controls metamorphic carbon release during mountain building and regulates the inorganic carbon budget, according to carbon estimates in two river catchments of Italy’s central Apennines.

## Main

Global plate motions impact Earth’s carbon cycle by modulating both the release of CO_2_ from the crust and mantle^1^ and the emission or sequestration of CO_2_ from rock weathering^2^. In uplifting mountains, near-surface (critical-zone) chemical weathering reactions are particularly efficient^3^, resulting in timescale-dependent changes in the CO_2_ content of the atmosphere through silicate and carbonate mineral weathering with carbonic acid (H_2_CO_3_) and sulfuric acid (H_2_SO_4_). Thus, orogenesis is proposed to impact global climate by increasing the weatherability of Earth’s surface^4,5^. However, mountain building can also generate large volumes of ‘metamorphic’ CO_2_ from the decarbonation or melting of carbonate in the crust and mantle^1,6–8^, where orogenesis involves the collision and subduction of carbonate rock. This release of CO_2_ ultimately reflects the conversion of carbonate to weatherable silicate minerals that completes the global silicate weathering cycle^9^. However, the different timescales required for metamorphic CO_2_ release and silicate weathering suggest mountain building may impact global climate through the modulation of these deep CO_2_ emissions^10,11^.

Determining the role of orogenesis in the global carbon cycle requires direct comparisons of its impact on both deep processes and chemical weathering. To our knowledge, such comparisons exist for only two locations. In the New Zealand Southern Alps, collision of mostly siliceous rocks results in degassing-related CO_2_ emission fluxes, which are an order of magnitude smaller than inferred CO_2_ drawdown fluxes from silicate weathering^12^. In the Himalaya, the subduction and collision of carbonates lead to CO_2_ degassing that outpaces silicate weathering fluxes^13,14^. These studies estimate metamorphic CO_2_ degassing directly from samples in springs, aquifers and gas vents, whereas weathering fluxes are estimated from riverine fluxes. Therefore, these datasets cannot constrain how deep, crustal or mantle-derived CO_2_ fluxes interact with the critical zone and may be buffered by carbonate and silicate weathering. Moreover, it remains unclear how geodynamics—the influence of mantle convection on tectonics—impact the relative spatial importance of degassing and weathering fluxes across a mountain range.

To address this important knowledge gap in the inorganic carbon cycle, we investigate the relationship between deep CO_2_ release and chemical weathering in the critical zone along a geodynamic gradient in the central Apennines (Italy), an active mountain range that exposes and subducts large volumes of carbonate. We present major riverine element and isotope data from two large catchments that straddle a gradient in crustal thickness and heat flow above a tear and area of retreat within the subducting slab and assess inorganic CO_2_ emission and sequestration fluxes associated with critical zone and deep processes. We use an inverse approach to apportion the solute flux to the weathering of carbonates, silicates and sulfide and to distinguish atmospheric, lithologic and metamorphic CO_2_ sources. The results of this study demonstrate that the inorganic carbon budget of the central Apennines is controlled primarily by metamorphic release that varies strongly across the regional geodynamic gradient. Hence, the impact of regional tectonics on CO_2_ sources may be substantially larger than tectonic modulation of CO_2_ sinks.

## Tectonic setting of the central Apennines

**Fig. 1:**
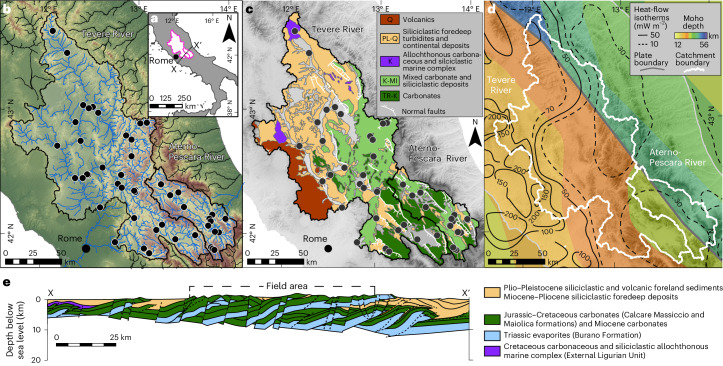
Overview of the sampling locations and the geologic and geodynamic setting. **a**, Location of studied catchments (pink outlines) and location of the cross-section shown in **e**. **b**, Sample distribution (black circles) and river networks. Networks for the studied rivers are shown in blue, and adjacent river networks are shown in black. **c**, Simplified geologic map. **d**, Geodynamic setting for the central Apennines. Moho depth is illustrated as a colour gradient. Heat-flow isotherms are illustrated as contour lines for a 10 mW m^–2^ contour interval (dashed lines) or a 50 mW m^–2^ contour interval (solid line) unless otherwise noted. **e**, Geologic cross-section through the Italian peninsula. The cross-section illustrates the ages, lithologies and major tectonic structures in the upper 20 km of the crust. Layers in **b–d** are overlain on a Shuttle Radar Topography Mission (SRTM) 90 m hillshade and digital elevation model. All individual sample points are shown in Extended Data Fig. 1. Panels adapted with permission from: **c**, refs. ^22,23^ under a Creative Commons license CC BY 3.0; **d**, ref. ^46^, Oxford University Press; ref. ^17^, Springer; ref. ^8^, Elsevier; **e**, ref. ^15^, The Virtual Explorer Pty Ltd.

The Apennine chain is a fold-and-thrust belt characterized by an accretionary wedge to the east and a back-arc extensional basin to the west^15^ (Fig. 1 and Supplementary Text 1), which developed through syn-convergent extension, due to the subduction and rollback of the Adriatic slab beneath Eurasia^16^. This dynamic has produced a tectonic gradient expressed by an increase in extension from east to west, resulting in lower crustal thickness (~20 km) and higher heat flow (>200 mW m^–2^) in the west relative to thicker crust (>40 km) and lower heat flow (<30 mW m^–2^) in the east^8,17,18^ (Fig. 1d). In the central Apennines, the absence of intermediate seismicity and the presence of anomalously low P-wave velocities have been interpreted as a slab window^8,19,20^ formed due to progressive east-directed rollback and tearing of the Adriatic slab. By contrast, intermediate and deep seismicity beneath the northern Apennines and Calabria illustrate an intact, subducting slab^20^.

## Water chemistry of the Tevere and Aterno-Pescara rivers

**Extended Data Fig. 1:**
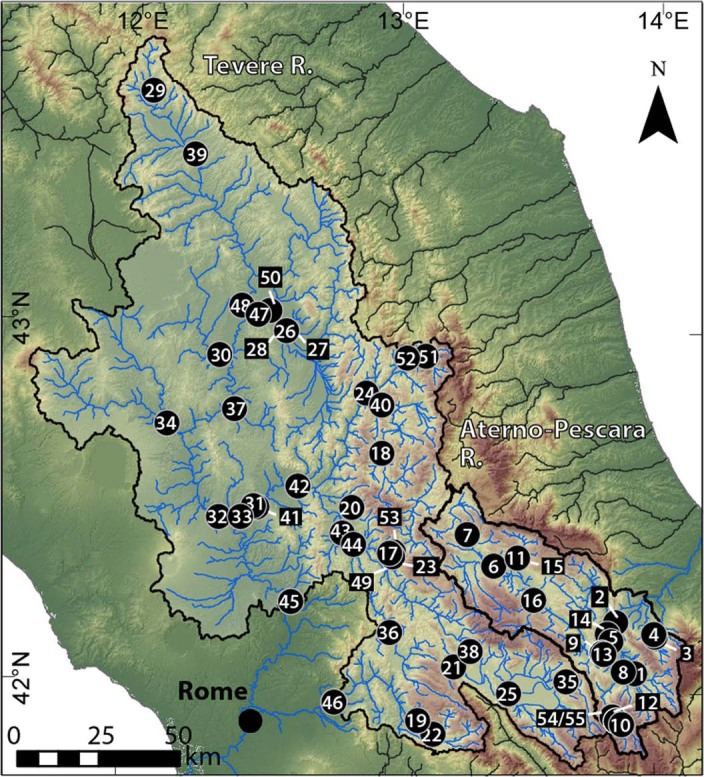
Location map with numbered sampling locations. Stream networks for the studied rivers are shown in blue, and adjacent stream networks are shown in black. Layers are overlain on a Shuttle Radar Topography Mission (SRTM) 90-m hillshade and digital elevation model (DEM).

We present 104 water samples collected during winter 2021 (55) and summer 2020 (49) from these catchments (Fig. 1 and Extended Data Fig. 1), which were selected to maximize areal coverage and to sample different lithologies and water bodies (for example, river, springs/groundwater, lakes), to understand the potential sources of dissolved ions to the river channels. For all samples, we measured concentrations of dissolved major elements as well as isotopes of inorganic carbon (δ^13^C, F^14^C (fraction modern carbon)) and sulfur and oxygen in sulfate (δ^34^S, δ^18^O(SO_4_)). On the basis of these measurements, we can unmix the contributions to the dissolved load of carbonate, silicate and evaporite mineral sources (Methods). In addition, we distinguish the acid sources for weathering, including sulfuric acid (H_2_SO_4_), carbonic acid (H_2_CO_3_) derived from biogenic or atmospheric CO_2_ and H_2_CO_3_ derived from metamorphic carbon.

**Fig. 2:**
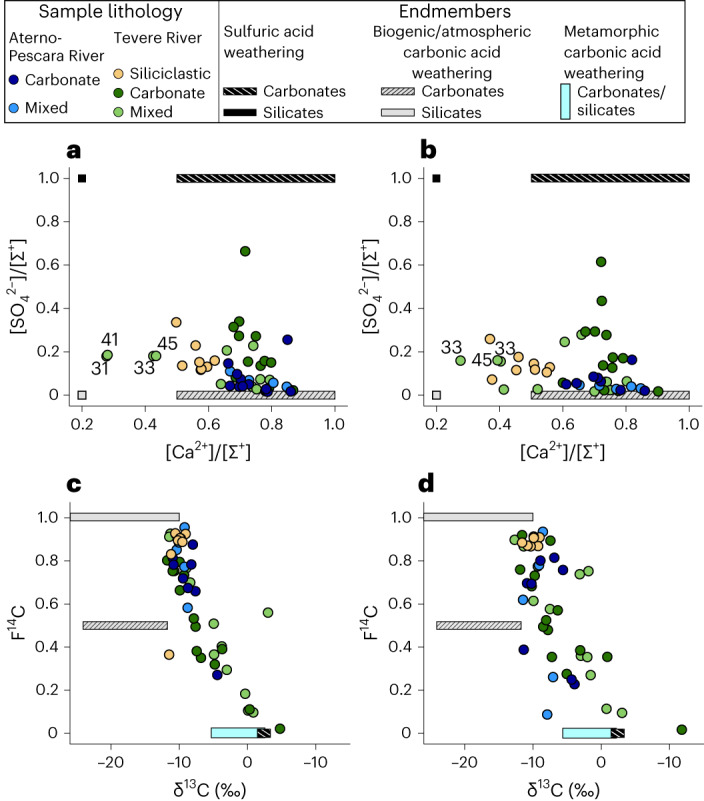
Water chemistry in relation to the expected chemical signatures of critical-zone weathering and metamorphic carbon. **a**,**b**, Ratios of [Ca^2+^]/[Σ^+^] plotted against ratios of [SO_4_^2−^]/[Σ^+^] for winter samples (**a**) and summer samples (**b**). **c**,**d**, δ^13^C plotted against F^14^C for winter samples (**c**) and summer samples (**d**). Samples are coloured by the dominant lithology in the upstream area^21^: carbonate, mixed carbonate and siliciclastic, or siliciclastic. Bars represent ion or isotopic endmember compositions^21^. Annotated samples in **a** and **b** are samples with low [Ca^2+^]/[Σ^+^] that have higher [Na^+^] than [Ca^2+^] and reflect evaporite δ^34^S and δ^18^O(SO_4_) signatures.

Because the weathering of carbonate versus silicate rocks can have different implications for the inorganic carbon cycle, we broadly categorize the lithology at each sampling location as ‘carbonate’, ‘siliciclastic’ or ‘mixed’ (a carbonate-siliciclastic mix)^21^ on the basis of the distribution of surface lithologies in refs. ^22,23^. The weathering of silicate or carbonate rock by H_2_CO_3_ or H_2_SO_4_ co-determines the resulting production of ions and dissolved inorganic carbon (DIC)^24^. In the absence of a gypsum contribution, the ratios of [SO_4_^2−^]/[Σ^+^] and [Ca^2+^]/[Σ^+^] (where Σ^+^ is the sum of cations) reflect the balance of carbonate and silicate weathering with a mixture of H_2_CO_3_ and H_2_SO_4_ sources. Most river samples have ratios of [SO_4_^2−^]/[Σ^+^] below 0.45 and [Ca^2+^]/[Σ^+^] ratios between ~0.3 and 0.9 (Fig. 2a,b). The siliciclastic samples and a subset of numbered Tevere samples collected at or near springs display the lowest [Ca^2+^]/[Σ^+^] ratios. Cations in these samples are dominated by Na^+^, rather than Ca^2+^, and the similarity between [Na^+^] and [Cl^−^] in these samples suggests that halite is probably the primary source of [Na^+^] (ref. ^21^). Overall, the high [Ca^2+^]/[Σ^+^] and low [SO_4_^2−^]/[Σ^+^] values of the samples suggest that the study area is dominated by H_2_CO_3_ weathering of carbonate and silicate rock.

**Extended Data Fig. 2:**
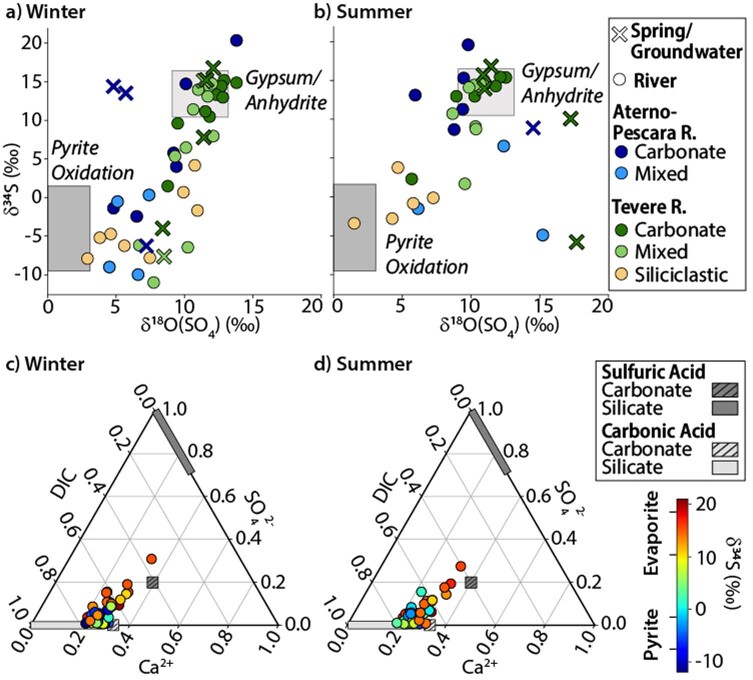
Endmember mixing models for sulfur, oxygen, and carbon isotopic signatures. δ^18^O(SO_4_^2−^) plotted against δ^34^S for (**a**) winter samples and (**b**) summer samples. Ternary diagram illustrating the proportion of measured DIC, SO_4_^2−^, and Ca^2+^ concentrations relative to endmember weathering acids for (**c**) winter samples and (**d**) summer samples. River samples are colored by the measured δ^34^S signature. **(a**, **b**) Samples are colored by surface lithology (carbonate, mixed, and siliciclastic) and by catchment (Aterno-Pescara or Tevere River). River and spring samples are illustrated as circle and x symbols, respectively, and the numbered springs correspond with sampling locations given in ref. ^21^. Endmember isotopic signatures for pyrite and evaporites are given in ref. ^21^.

Carbon isotopes and major element geochemistry yield insights into the proportions of DIC sourced from modern carbon (F^14^C = 1; for example, biogenic or atmospheric carbon) and from rock-derived, radiocarbon dead sources (F^14^C = 0; for example, from carbonate weathering or metamorphic carbon). Both δ^13^C and F^14^C values are highly variable and reflect these different carbon sources (Fig. 2c,d). Most samples lie between the biogenic (modern) carbon and the carbonate–H_2_CO_3_ endmembers, while approximately 30% of samples lie beyond the carbonate–H_2_CO_3_ endmember. These low F^14^C and enriched δ^13^C values could be explained by H_2_SO_4_ dissolution of carbonates^25^; however, major element chemistry is inconsistent with such large contributions of H_2_SO_4_ to the weathering budget (Extended Data Fig. 2). Hence, the low F^14^C and enriched δ^13^C values require that a substantial proportion of the H_2_CO_3_ is derived from upwelling of deeper, rock-derived CO_2_-rich fluids^8^.

Studies in the central Apennines from the past two decades have identified δ^13^C-enriched sources of CO_2_ at cold and thermal mineralized springs^26–28^ and in the regional aquifers^29^, as well as CO_2_ degassing from localized gas vents. The geochemical (^4^He/^3^He) signature of CO_2_ emissions suggests that degassing fluxes are sourced predominantly from melting of the carbonate sedimentary cover on the subducting Adriatic slab within the mantle lithosphere^8,30,31^, producing carbonate-rich melts that upwell through the mantle^8^. Additional CO_2_ derives from decarbonation of carbonates in the overriding Eurasian plate^30^. Localized metamorphic CO_2_ outgassing has been linked with periods of high seismicity^16,32,33^, suggesting that widespread normal faults and fractures are effective conduits for CO_2_-rich fluids that migrate through the crust^34^ (Fig. 1c,e). On reaching the surface, the CO_2_ either is outgassed at vents^28^ or mixes with meteoric water in the regional carbonate aquifers and can be released at springs^8,35,36^. Our chemical analysis of the stream waters suggests that CO_2_ not only is directly degassed but effectively interacts with the critical zone by providing H_2_CO_3_ that can weather carbonate and silicate rocks near the surface.

## A CO_2_ budget for the central Apennines

To quantitatively deconvolve the sources of DIC and contributions of lithologic endmembers to central Apennine rivers, we use a recent inverse model, Mixing Elements and Dissolved Isotopes in Rivers (MEANDIR)^37^. We quantify the fraction of major ions (Ca^2+^, Na^+^, Mg^2+^, SO_4_^2+^, Cl^−^) contributed from silicates, carbonates, evaporites, pyrite oxidation and meteoric water, as well as the fraction of DIC from biogenic carbon, rock-derived carbon, and atmospheric carbon and meteoric water (Methods). The pyrite oxidation endmember allows us to quantify the proportion of weathering by H_2_SO_4_. Together with the relative proportions of biogenic and rock-derived carbon, it further allows us to constrain the fraction of carbon derived from deep sources. Where possible, we convert ion concentrations to fluxes by multiplying molar masses of the respective ion with ion concentrations and available run-off estimates averaged over the months of data collection (Methods). We note that our model inputs of Ca^2+^, HCO_3_^−^ and δ^13^C are corrected for the effects of secondary carbonate precipitation, which accounts for the loss of 45% of [Ca^2+^] for locations included in our carbon budget^21^. After this correction, estimates of [DIC] increase by 0–181% ([DIC]_Corr_) and metamorphic CO_2_ fluxes are 0–45% higher^21^.

**Fig. 3:**
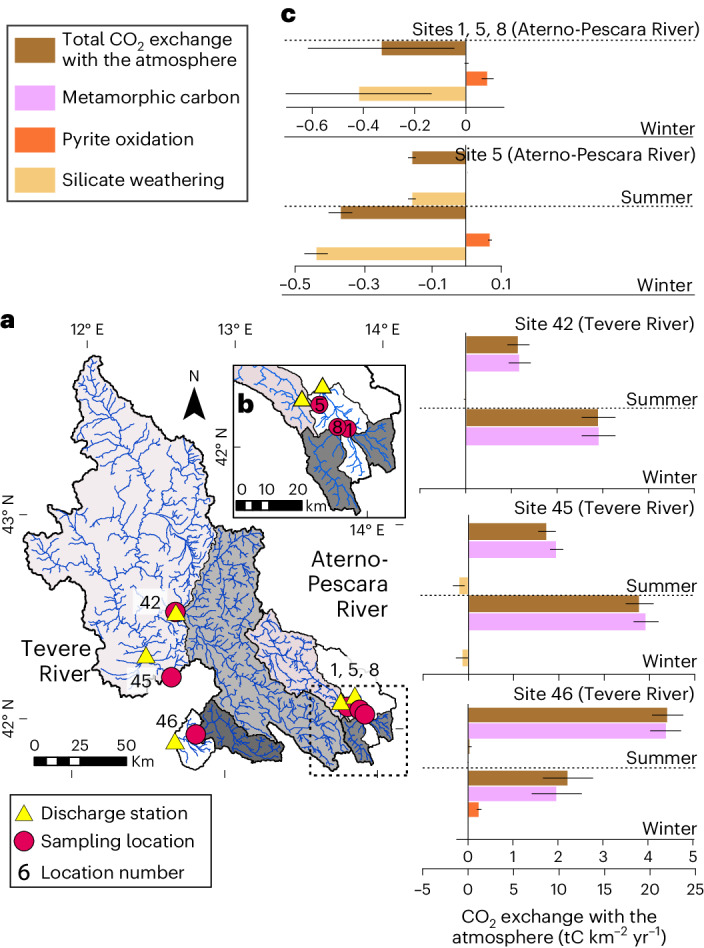
CO_2_ sinks and sources inferred from the Tevere and Aterno-Pescara River chemistry. Regional inorganic CO_2_ fluxes from five sampling locations with the largest upstream drainage area. **a**, The sampling locations, site numbers^21^ and closest discharge stations. Upstream catchment areas from each sampling location are outlined in black and coloured in grey, and the corresponding drainage network (blue lines) is shown. Note that the upstream area for site 45 also includes the area encompassed by site 42. **b**, Enlarged image of Aterno-Pescara sampling locations and discharge stations. **c**, CO_2_ exchange with the atmosphere, shown as fluxes associated with mechanisms that result in long-term CO_2_ drawdown (silicate weathering) or CO_2_ release (pyrite oxidation and metamorphic carbon). The sum of the fluxes is illustrated as the total CO_2_ exchange with the atmosphere. Data bars represent mean values ± 1*σ* (ref. ^21^).

**Extended Data Fig. 3:**
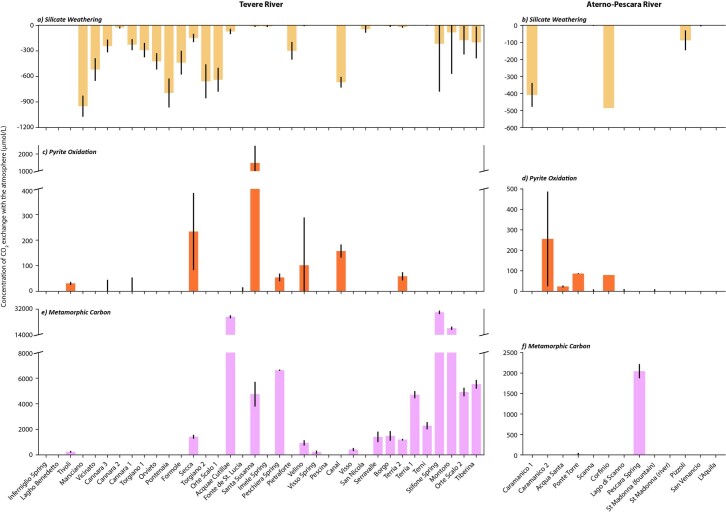
CO_2_ sinks and sources (winter samples) illustrated in concentration units. CO_2_ exchange with the atmosphere is attributed to (**a**) and (**b**) silicate weathering, (**c**) and (**d**) pyrite oxidation, and (**e**) and (**f**) metamorphic carbon for sampling locations along the Tevere River (left column) and the Aterno-Pescara River (right column). Data bars represent sample mean values ± 1σ (ref. ^21^).

**Extended Data Fig. 4:**
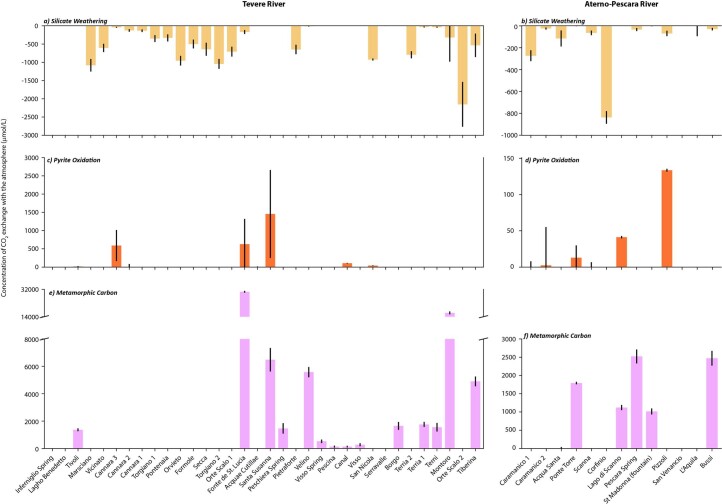
CO_2_ sinks and sources (summer samples) illustrated in concentration units. CO_2_ exchange with the atmosphere is attributed to (**a**) and (**b**) silicate weathering, (**c**) and (**d**) pyrite oxidation, and (**e**) and (**f**) metamorphic carbon for sampling locations along the Tevere River (left column) and the Aterno-Pescara River (right column). Data bars represent sample mean values ± 1σ (ref. ^21^).

**Extended Data Fig. 5:**
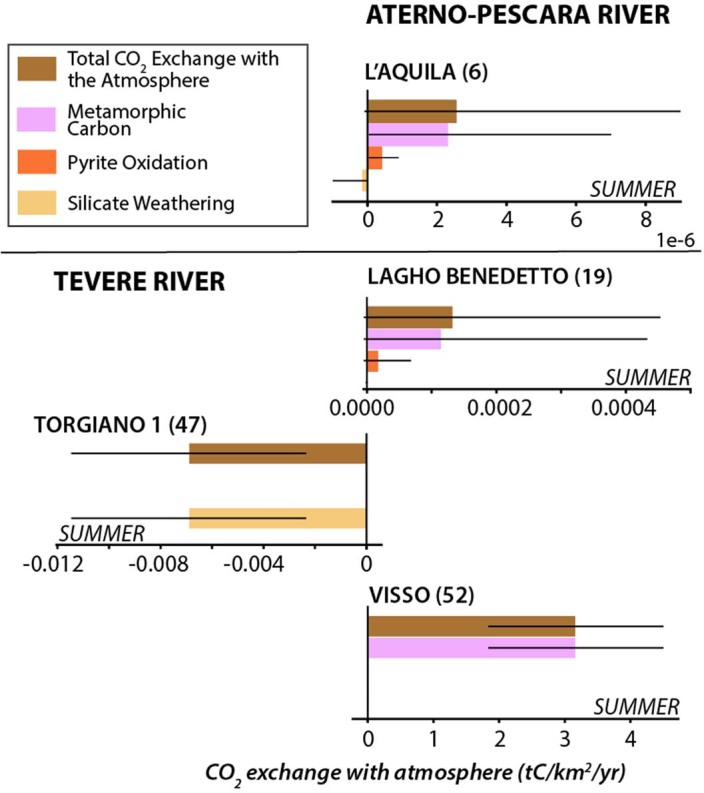
CO_2_ sinks and sources inferred from the Tevere and Aterno Pescara River chemistry for all locations with successful MEANDIR runs using the ‘iterate over samples’ approach and available discharge measurements. CO_2_ exchange with the atmosphere is shown as fluxes associated with mechanisms that result in long-term CO_2_ drawdown (silicate weathering) or CO_2_ release (pyrite oxidation and metamorphic carbon). The sum of the fluxes is illustrated as the total CO_2_ exchange with the atmosphere. Data bars represent mean values ± 1σ (ref. ^21^). Note that the fluxes from L’Aquila are shown to illustrate the dominate flux patterns, although the fluxes themselves are on the order of 1e^−6^ and are thus negligible.

**Extended Data Fig. 6:**
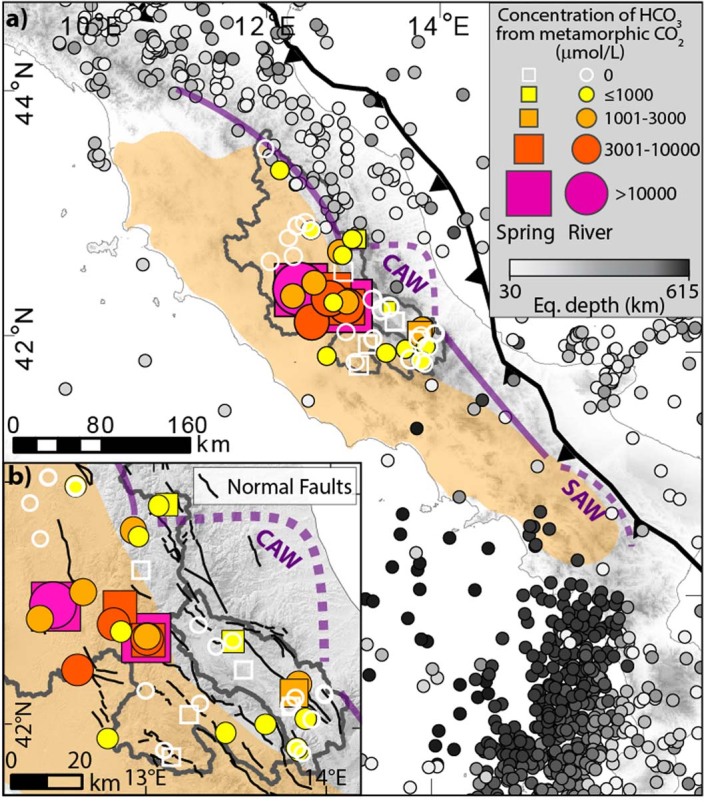
Overview of the regional geodynamic setting in relation to the full set of metamorphic CO2 results from both the ‘iterate over samples’ and ‘iterate over endmembers’ model approaches. a) and b) Colored circles and squares illustrate samples with non-zero yearly average HCO_3_ from metamorphic carbon, scaled by the concentration. White, outlined circles and squares illustrate samples where HCO_3_ from metamorphic CO_2_ is zero (ref. ^21^). The purple line marks the boundary between low p-wave velocity (V_p_) anomalies to the west and high V_p_ anomalies to the east at 52 km depth, and the dashed indentations in the line mark the locations of slab windows in the central (CAW) and southern Apennines (SAW). The orange overlay illustrates the spatial extent of measured CO_2_ gas emissions, gray circles illustrate the locations and depth of seismicity deeper than 40 km and magnitude 3 or higher, and the black sawtoothed line marks the subduction front. b) illustrates an enlarged view of the results within the study area, including the locations of normal faults (black lines). Figure adapted with permission from refs. ^8,47,48^ Elsevier.

We follow previous work^38^ and infer CO_2_ sequestration and release from our fluxes on timescales longer than the compensation of alkalinity fluxes to the ocean by carbonate precipitation (1–10 kyr) (ref. ^39^) but shorter than the timescales for sulfur reduction in the ocean (>10 Myr)^39^. We find that the solute and carbon budget of the main rivers in the study area are variable in space and related to the geomorphic setting. In the Aterno-Pescara River, CO_2_ fluxes (reported in tons of carbon (tC) per area per time) are dominated by silicate weathering (−0.4–0 tC km^–2^ yr^–1^), with minor fluxes from coupled pyrite oxidation and carbonate weathering (0–0.1 tC km^–2^ yr^–1^) but no measurable metamorphic carbon fluxes (Fig. 3), with the exception of springs or small tributaries near or along faults (Extended Data Figs. 3–6). CO_2_ fluxes from pyrite oxidation (0–0.2 tC km^2^ yr^–1^) and silicate weathering (−1.1–0 tC km^2^ yr^–1^) in the Tevere River are of similar magnitude to the Aterno-Pescara River. However, the net CO_2_ fluxes in the Tevere are 1–2 orders of magnitude higher than in the Aterno-Pescara River due to large CO_2_ fluxes inferred from metamorphic carbon (Fig. 3). In the largest Tevere tributaries, the flux of metamorphic CO_2_ is consistently 1−2 orders of magnitude higher than fluxes from silicate weathering and pyrite oxidation, respectively (Fig. 3)^21^. While smaller tributaries that drain siliciclastic-rich lithologies are dominated by silicate weathering (Extended Data Figs. 3–6), the regional inorganic carbon budget shows that the central Apennines are a net carbon source.

Our field seasons broadly reflect the hottest and driest or wettest and coldest times of the year^40^, allowing us to estimate minimum (summer) and maximum (winter) yearly CO_2_ fluxes. We calculate a minimum net CO_2_ flux of 7.9 ± 2.4 tC km^–2^ yr^–1^ over an area of 18,243 km^2^ (Sites 5, 45 and 46) and a maximum estimate of 16.4 ± 6.3 tC km^–2^ yr^–1^ over an area of 18,655 km^2^ (Sites 1, 5, 8, 45 and 46). Overall, the weighted yearly average net CO_2_ flux is 12.3 ± 4.1 tC km^–2^ yr^–1^ (Sites 5, 45 and 46). Relative to a yearly metamorphic CO_2_ flux upscaled from spring data (28 tC km^–2^ yr^–1^) (ref. ^29^), our estimate of CO_2_ fluxes is about a factor of 2 lower. These two values probably constitute minimum and maximum estimates, respectively, and their difference could be due to two reasons. First, our river estimates may underestimate CO_2_ fluxes because they miss rapid, diffusion-controlled CO_2_ degassing that is not associated with secondary precipitation of carbonate and has a negligible effect on the carbon isotopic composition of the water^41^. Second, upscaled fluxes from springs to the entire watersheds could overestimate the regional flux of CO_2_, because they miss diffuse inputs of water across the catchments that can represent between ~20% and 100% of riverine major ion concentrations^42^. Despite these uncertainties, we can conclude that CO_2_ fluxes from metamorphic carbon in the Tevere River are orders of magnitude larger than CO_2_ drawdown fluxes from silicate weathering in these watersheds.

**Fig. 4:**
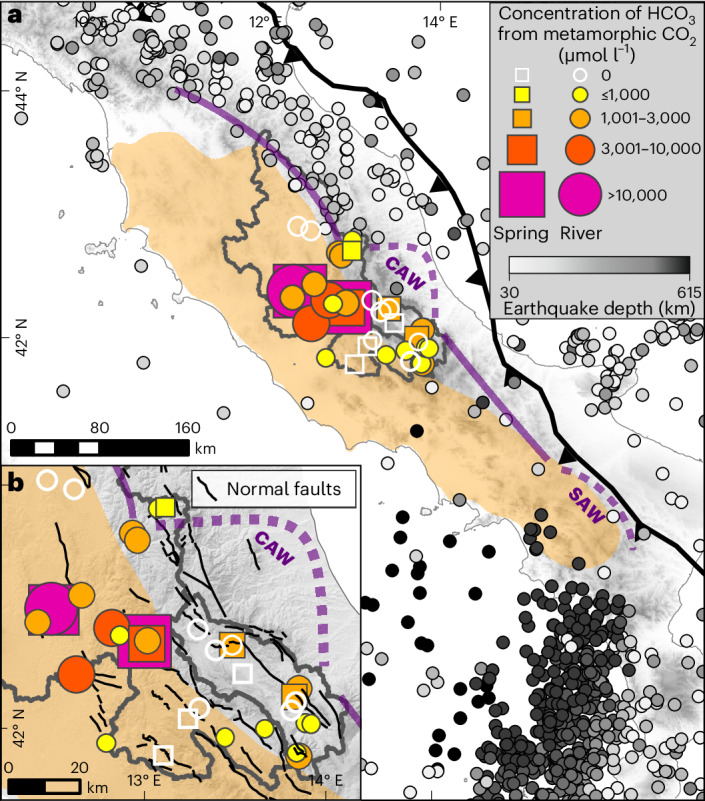
Overview of the regional geodynamic setting in relation to the metamorphic CO_2_ contributions to spring and stream waters. **a**, Coloured circles and squares illustrate samples with non-zero yearly average HCO_3_ from metamorphic carbon, scaled by the concentration^21^. Where both winter and summer concentrations were available for a sample, the symbol represents an average over the two seasons. White-outlined circles and squares illustrate samples where HCO_3_ from metamorphic CO_2_ is zero. The purple line marks the boundary between P-wave velocity (*V*_p_) anomalies at 52 km depth; CAW and SAW on the dashed portion of the line highlight the locations of slab windows in the Central and Southern Apennines, respectively. The orange overlay illustrates the spatial extent of measured CO_2_ gas emissions, grey circles illustrate the locations and depths of seismicity deeper than 40 km and magnitude 3 or higher, and the black sawtoothed line marks the subduction front. **b**, Enlarged view of the results within the study area, including the locations of normal faults (black lines). Figure adapted with permission from refs. ^8,47,48^, Elsevier; ref. ^49^ under a Creative Commons license CC BY 4.0.

To estimate a total carbon budget for the Apennines, we combine our results with estimates for inorganic CO_2_ emissions from gas vents and organic CO_2_ exchanges^2^. Discrete CO_2_ emissions from gas vents reported on the western side of the Apennines contribute 2–12 tC km^–2^ yr^–1^ over an estimated area of 52,000 km^2^ (Fig. 4)^8,36^. Estimates for petrogenic organic carbon oxidation do not exist for the Apennines but are probably small in these lithologies—analogous to the small sulfide oxidation rates. Particulate organic carbon export at 200 m depth in the Tyrrhenian Sea ranges from 1.3 to 6.1 tC km^–2^ yr^–1^ for spring and summer and is 0.7 tC km^–2^ yr^–1^ in the Adriatic Sea during autumn^43^. Estimates for dissolved organic carbon burial are lacking, but dissolved organic carbon export is probably much smaller than the particulate organic carbon export^44^. The central Apennines are thus a net carbon source on the western side of the mountain range, where CO_2_ emissions from metamorphic decarbonation are 2–10 times larger than organic carbon burial and 1–2 orders of magnitude larger than CO_2_ drawdown from silicate weathering. On the eastern side, the inorganic CO_2_ budget is dominated by silicate weathering and CO_2_-releasing sulfide oxidation from carbonate weathering, although the magnitude of carbon sources is much smaller relative to the western side of the range, so they may be compensated by organic carbon burial.

## Impact of geodynamic setting on the inorganic CO_2_ budget

**Fig. 5:**
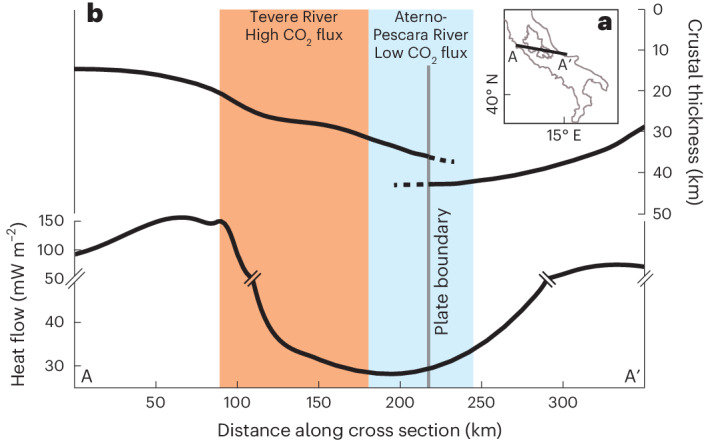
Schematic cross-section through the Central Apennines illustrating major patterns in CO_2_ fluxes, heat flow, and crustal thickness. The east–west patterns of CO_2_ fluxes, heat flow^8,17^ and Moho depth^46^ across the Central Apennines. **a**, The location of the cross-section from A to A′ is shown in the inset. **b**, The intersections of the heat flow and Moho depth data with the locations of the Tevere and Aterno-Pescara catchments are illustrated with orange and blue shaded areas, respectively.

The stark difference in both the sources of CO_2_ and the magnitude of CO_2_ fluxes between the Tevere (Tyrrhenian side) and Aterno-Pescara Rivers (Adriatic side) coincides with a regional east to west geodynamic and tectonic gradient defined by a westward increase in extension and heat flow and a decrease in crustal thickness^8,17,18^ (Figs. 1d, 5). In contrast, climatic and lithologic differences between the two river systems are small. We propose that thin crust with high heat flow in the Tevere River drives important release of metamorphic CO_2_ (Figs. 3–5). In turn, higher crustal thickness and lower heat flow in the Aterno-Pescara River inhibit substantial CO_2_ release. Here only springs and river samples along or near faults provide evidence for metamorphic CO_2_ release (Fig. 4), and the composition of catchment-averaged river samples of the Aterno-Pescara River can be explained without any metamorphic inputs. The geodynamic control on CO_2_ release is also evident from the pattern of metamorphic CO_2_ emissions measured from local gas vents^36^ that are primarily on the Tyrrhenian side of the mountain range or are found almost exclusively along faults on the Adriatic side of the range (Fig. 4). This area coincides with the location of the slab window (CAW) (Fig. 4), whereas CO_2_ emissions have not been reported in Calabria and in the northern Apennines, where a subducting slab is still intact. Slab retreat and break-off have acted as a catalyst for regional mantle convection and increased heat flow^19,20,16^, which in turn facilitated melting and decarbonation of the carbonate sedimentary cover on the Adriatic plate^8^. Mantle upwelling induced by the slab dynamics is also responsible for driving long-wavelength uplift^19,16^, which in turn activated the extensional structures that bring metamorphic CO_2_ to the surface^34^. Due to the apparent link between the location of CO_2_ release with slab retreat and tearing, the timing of slab detachment (~2 Ma)^20^, and normal fault activation (2.5 and 3.3 Ma), we suggest that the dominance of metamorphic CO_2_ release on the Tyrrhenian side—and potentially the east–west contrast in the inorganic carbon budget—may have been present over at least 2 Ma.

The large differences in the riverine solute budget between the two major Apennine river systems, the location of reported CO_2_ gas emissions and the absence of major climatic or lithologic gradients across the study area support the notion that differences in crustal thickness and heat flow could cause order-of-magnitude variations in inorganic CO_2_ cycling over length scales of a few tens of kilometres (Fig. 5). Importantly, the variations in the flux of metamorphic CO_2_ release are much larger than variations in chemical weathering fluxes across the study area. Thus, in the central Apennines, the regional geodynamics and tectonics impact mountain building and the carbon cycle most significantly by modulating the release of metamorphic CO_2_, not by enhancing CO_2_ drawdown or release from critical-zone weathering reactions^45^. Furthermore, the subduction of passive margin carbonate-rich sediments and extension-induced variations in heat flow and crustal thickness reflect orogenic processes common to other mountain ranges during the initial stages of orogenesis^1^. We suggest that modelling and understanding the true impact of early-stage mountain building on the global carbon cycle should consider the broader role of geodynamics and tectonics beyond uplift and the balance of critical-zone weathering reactions.

## Methods

### Ion and isotope measurements

For each sample location, we collected water in high-density polyethylene bottles for cations (30 ml), anions (60 ml), alkalinity (250 ml) and δ^34^S and δ^18^O(SO_4_^2−^) (250 ml) analyses. We filtered water samples in the field with 0.2 μm VWR filters and acidified cation samples with two drops of ultra-pure 36% HNO_3_. We also collected water in glass vials for δ^13^C and δ^14^C (20 ml) analyses. We measured the alkalinity of each sample within 24 hours of collection using Gran Titration with a Hach digital titrator. All samples were kept at the German Research Centre for Geosciences (GFZ) in cold storage at 4 °C before analysis.

To deconvolve the major lithologies being weathered in the Central Apennines, we measured the concentrations of major dissolved ions for each water sample. We measured major cations (Ca^2+^, K^+^, Mg^2+^, Na^+^) and dissolved silica on a Varian 720ES inductively coupled plasma optical emission spectroscope at the GFZ. To monitor machine drift, quality control samples were run for every ten measured samples, and accepted runs had a drift of <5%. We performed quality control tests using water standards SLRS-6 and USGS-T187. A set of 11 in-house standards were used to calibrate the cation measurements, and only those standards that fell within 10% of a linear fit through all standards were accepted. Similarly, we accepted only cation measurements within the range of the accepted standards. Measurement uncertainty was estimated from the maximum deviation of the calibration standards from the calibration line.

We measured major anions (F^-^, Cl^−^, SO_4_^2−^) at the GFZ on a Dionex ICS-1100 chromatograph. Quality control was performed using a six-point linear calibration and USGS-206 and USGS-212 standards. We quantified measurement uncertainty on the basis of the standard deviation of three repeat measurements.

To distinguish between lithologic sources of riverine sulfate, we measured δ^34^S and δ^18^O(SO_4_) at the Centre de Recherche Pétrographiques et Géochimiques. The δ^34^S samples were prepared according to ref. ^50^. We extracted anions from water samples using column chemistry, with cationic resin AG1X8, and subsequently dried down and diluted the samples in 5% HNO_3_. We measured δ^34^S using a Thermo Fischer Scientific multicollector inductively coupled plasma mass spectrometer Neptune Plus (IRISS platform). Values are provided in the Vienna Canyon Diablo Troilite scale, thanks to an in-house bracketing standard calibrated against the International Atomic Energy Agency (IAEA) S1 standard^51^. External reproducibility is based on independently purified replicates of seawater, which had average measured δ^34^S values of 21.2 ± 0.12‰ (summer) and 21.2 ± 0.05‰ (winter), reported with 2*σ* errors.

We prepared δ^18^O(SO_4_) samples by measuring 250 ml of river water and acidifying the solution with HCl 3 N to a pH of 4.2 to eliminate HCO_3_ and CO_3_. The initial 250 ml of water was then heated to 200 °C for 30 minutes to eliminate CO_2_. The temperature was then adjusted to 70 °C, and a 5% BaCl_2_ solution was added to the water in a volume proportional to the measured concentration of SO_4_ in the individual water sample. The BaSO_4_ was allowed to precipitate from the solution for 1 hour, and then overnight at the ambient temperature. The precipitate was filtered using a 0.2 μm Nylon filter and subsequently rinsed twice with distilled water and three times with acidified water (5 ml HCl per litre water). The precipitate was then dried at 100 °C. We measured the δ^18^O(SO_4_) on a Thermo Fisher EAlsolink-Delta V isotope ratio mass spectrometer. Measured standards yielded δ^18^O(SO_4_) values of 23.3 ± 0.4‰ (IAEA) and 9.3 ± 0.4‰ (NBS 127) for the winter sample set, and 23.3 ± 0.8‰ (IAEA) and 9.3 ± 0.6‰ (NBS 127) for the summer sample set (reported with 2*σ* errors). We note that some of the samples lack replicates from each season (labelled as ‘NA’) because there was insufficient BaSO_4_ to measure δ^18^O(SO_4_) (ref. ^21^).

To distinguish between atmospheric and rock sources of dissolved carbon, we measured DI^13^C and DIC concentrations at the Centre de Recherche Pétrographiques et Géochimiques and DI^14^C at the Laboratory for Ion Beam Physics at the ETH Zürich. For δ^13^C measurements, the samples and H_3_PO_4_ were put into glass vials and vaporized. The isotopic composition of the dissolved inorganic carbon within the remaining CO_2_ gas was measured with a Thermo Fisher EAlsolink-Delta V isotope ratio mass spectrometer. The DIC concentrations were estimated relative to a pure, synthetic calcite internal standard. We assumed conservative errors of 10% on the DIC calculations due to differences in the volume of air within the sampling tubes between liquid and solid samples (estimated to be 5%). The δ^14^C samples were prepared by purging 6 ml aliquots of each water sample with helium, acidifying the sample with 150 μl of 85% H_3_PO_4_ and heating the sample to 60 °C for 2 hours. We measured δ^14^C from the CO_2_ gas that formed from this process using an online carbonate handling system, which was connected to a mini carbon accelerator mass spectrometer (MICADAS AMS) equipped with a gas-accepting ion source.

### Weathering reactions: theoretical expectations

We assume silicate and carbonate compositions equivalent to the generic endmembers normalized by Σ^+^ (refs. ^37,21^) and converted to units of mol/mol.

Within this framework, we constrain endmember compositions for carbonic acid (H_2_CO_3_) and sulfuric acid (H_2_SO_4_) weathering of carbonate and silicate rock (Fig. 2 and equations (1)−(4)).

1 $${{\mathrm{CaSi}}}{{\mathrm{O}}}_{3}+2{{\mathrm{H}}}_{2}{\mathrm{C}}{{\mathrm{O}}}_{3}+{{\mathrm{H}}}_{2}{\mathrm{O}}\to {{{\mathrm{Ca}}}}^{2+}+{{\mathrm{H}}}_{4}{{\mathrm{Si}}}{{\mathrm{O}}}_{4}+2{{\mathrm{HC}}}{{\mathrm{O}}}_{3}^{-}$$

2 $${{\mathrm{CaC}}}{{\mathrm{O}}}_{3}+{{\mathrm{H}}}_{2}{\mathrm{C}}{{\mathrm{O}}}_{3}\to {{{\mathrm{Ca}}}}^{2+}+2{{\mathrm{HC}}}{{\mathrm{O}}}_{3}^{-}$$

3 $${{\mathrm{CaSi}}}{{\mathrm{O}}}_{3}+{{\mathrm{H}}}_{2}{\mathrm{S}}{{\mathrm{O}}}_{4}+{{\mathrm{H}}}_{2}{\mathrm{O}}\to {{{\mathrm{Ca}}}}^{2+}+{{\mathrm{H}}}_{4}{{\mathrm{Si}}}{{\mathrm{O}}}_{4}+{\mathrm{S}}{{\mathrm{O}}}_{4}^{-}$$

4 $${{\mathrm{CaC}}}{{\mathrm{O}}}_{3}+{{\mathrm{H}}}_{2}{\mathrm{S}}{{\mathrm{O}}}_{4}\to {{{\mathrm{Ca}}}}^{2+}+{{\mathrm{H}}}_{2}{\mathrm{O}}+{\mathrm{C}}{{\mathrm{O}}}_{2}+{\mathrm{S}}{{\mathrm{O}}}_{4}^{2-}$$

The weathering reactions in equations (1)–(4) also produce different theoretical DIC signatures^52^ that reflect modern carbon sources (for example, biogenic carbon or atmospheric carbon from meteoric water) and rock-derived, radiocarbon dead carbon sources (for example, rock and metamorphic carbon). The H_2_CO_3_ weathering of carbonate contributes 1 mole of modern carbon and 1 mole of rock-derived carbon to the solvent (water), so the expected fraction modern carbon (F^14^C) is 0.5, whereas silicate weathering by H_2_CO_3_ contributes only modern carbon and thus has an expected F^14^C signature of 1. Similarly, weathering 1 mole of carbonate minerals by H_2_SO_4_ contributes only rock-derived carbon (F^14^C = 0), whereas silicate weathering by H_2_SO_4_ produces no alkalinity or DIC since the reaction does not include any carbon species (equation (3)).

These theoretical expectations do not account for the dissolution of evaporites (for example, halite and gypsum). In the Tevere River, some spring and proximal river samples illustrate [Na^+^] that are higher than [Ca^2+^]. All other samples illustrate higher [Ca^2+^] than [Na^+^], even for samples derived from siliciclastic-rich turbidites. Assuming that all SO_4_^2−^ and Cl^−^ are derived from evaporite and balancing the cations, virtually all of the Na^+^ would be removed from solution for halite, but 7−98% of Ca^2+^ would be removed. Thus, correcting the samples for evaporite dissolution would have the overall effect of increasing Ca/Σ^+^ and decreasing SO_4_/Σ^+^.

### Elemental and isotope corrections for degassing and secondary carbonate precipitation

#### Elemental corrections for secondary carbonate precipitation

Secondary carbonate precipitation enriches the remaining fluid with Sr^2+^ so that Sr^2+^/Ca^2+^ increases. We can express the final Sr^2+^/Ca^2+^ ratio of the fluid relative to the initial ratio of Sr^2+^ to Ca^2+^ $\left(\frac{{\left[{{{\mathrm{Sr}}}}^{2+}\right]}_{0}}{{\left[{{{\mathrm{Ca}}}}^{2+}\right]}_{0}}\right)$, which reflects the absence of secondary precipitation:

5 $$\frac{[{{{\mathrm{Sr}}}}^{2+}]}{\left[{{{\mathrm{Ca}}}}^{2+}\right]}=\frac{{\left[{{{\mathrm{Sr}}}}^{2+}\right]}_{0}}{{\left[{{{\mathrm{Ca}}}}^{2+}\right]}_{0}}{{\gamma }_{{{\mathrm{CaC}}}{{\mathrm{O}}}_{3}}}^{({{\mathrm{kd}}}-1)}$$

Here, kd is the partition coefficient for Sr^2+^, and ${\gamma }_{{{\mathrm{CaC}}}{{\mathrm{O}}}_{3}}$ is the fraction of primary calcite that remains in the fluid^53^. The ${\gamma }_{{{\mathrm{CaC}}}{{\mathrm{O}}}_{3}}$ can range from 0 to 1, where a value of 1 reflects no loss of primary carbonate, and a value of 0 would theoretically indicate that all primary carbonate has been lost to secondary precipitation. We constrained $\frac{{\left[{{{\mathrm{Sr}}}}^{2+}\right]}_{0}}{{\left[{{{\mathrm{Ca}}}}^{2+}\right]}_{0}}$ from bedrock ratios in generic silicate and carbonate bedrock compositions^54^, which form an endmember mixing line expressed by:

6 $$\frac{1,000[{{{\mathrm{Sr}}}}^{2+}]}{\left[{{{\mathrm{Ca}}}}^{2+}\right]}=a+b\frac{\left[{{{\mathrm{Na}}}}^{+}\right]}{\left[{{{\mathrm{Ca}}}}^{2+}\right]}$$

where *a* and *b* are the intercept and slope of the fit, respectively. The amount of secondary carbonate precipitation is then calculated as the deviation of the solute samples from the bedrock mixing line. We can solve for $\frac{{\left[{{{\mathrm{Sr}}}}^{2+}\right]}_{0}}{{\left[{{{\mathrm{Ca}}}}^{2+}\right]}_{0}}$ by rearranging equation (5):

7 $$\frac{{\left[{{{\mathrm{Sr}}}}^{2+}\right]}_{0}}{{\left[{{{\mathrm{Ca}}}}^{2+}\right]}_{0}}=\frac{[{{{\mathrm{Sr}}}}^{2+}]}{\left[{{{\mathrm{Ca}}}}^{2+}\right]}{{\gamma }_{{{\mathrm{CaC}}}{{\mathrm{O}}}_{3}}}^{(1-{{\mathrm{kd}}})}$$

Assuming the concentration of Na^+^ does not change due to secondary carbonate precipitation, we can express the initial ratio of Sr^2+^/Ca^2+^ in terms of the Na^+^/Ca^2+^ ratio:

8 $$\frac{1,000{[{{{\mathrm{Sr}}}}^{2+}]}_{0}}{{[{{{\mathrm{Ca}}}}^{2+}]}_{0}}=a+b\frac{{\left[{{{\mathrm{Na}}}}^{+}\right]}_{0}}{{\left[{{{\mathrm{Ca}}}}^{2+}\right]}_{0}}=a+b\frac{\left[{{{\mathrm{Na}}}}^{+}\right]}{\left[{{{\mathrm{Ca}}}}^{2+}\right]}{\gamma }_{{{\mathrm{CaC}}}{{\mathrm{O}}}_{3}}$$

where $$\frac{{\left[{{{\mathrm{Na}}}}^{+}\right]}_{0}}{{\left[{{{\mathrm{Ca}}}}^{2+}\right]}_{0}}=\frac{\left[{{{\mathrm{Na}}}}^{+}\right]}{\left[{{{\mathrm{Ca}}}}^{2+}\right]}$$

We then combine equations (7) and (8) and solve for ${\gamma }_{{{\mathrm{CaC}}}{{\mathrm{O}}}_{3}}$ numerically:

9 $$\frac{1,000[{{{\mathrm{Sr}}}}^{2+}]}{[{{{\mathrm{Ca}}}}^{2+}]}{{\gamma }_{{{\mathrm{CaC}}}{{\mathrm{O}}}_{3}}}^{\left(1-{{\mathrm{kd}}}\right)}-a-b\frac{\left[{{{\mathrm{Na}}}}^{+}\right]}{\left[{{{\mathrm{Ca}}}}^{2+}\right]}{\gamma }_{{{\mathrm{CaC}}}{{\mathrm{O}}}_{3}}=0$$

We corrected for secondary precipitation with a partition coefficient of *k* = 0.05, which is within the acceptable range of values for *k* (0.02–0.20) (refs. ^55–57^).

#### Isotope fractionation due to CO_2_ degassing and secondary carbonate precipitation

Fractionation of bicarbonate in water happens during degassing of CO_2_ and during precipitation of CaCO_3_. We assess the effect of CO_2_ degassing and secondary carbonate precipitation on measured DIC and Ca^2+^ concentrations and on DI^13^C isotopic signatures. Reporting ^14^C as fraction modern (F^14^C) already accounts for fractionation that occurs in nature^58^, so we do not correct these values.

Degassing will preferentially result in the loss of more depleted carbon, thus enriching the remaining fluid, whereas precipitation of carbonate will result in the loss of heavier (more enriched) carbon. To correct for CO_2_ degassing and secondary precipitation, we use fractionation and enrichment factors that describe how carbon isotopes are fractionated due to individual processes^21^. Following the methodology of ref. ^59^, we assume (1) that CO_2_ degassing and secondary precipitation are irreversible reactions within an open system^13^ and (2) that half of the enrichment is due to degassing and half is due to precipitation, so that carbon is equally distributed between the two reactions. The carbon isotopic signature is expressed as ratio *R* (for example, ^13^C/^12^C) where the heavier isotope is the numerator. The fractionation of *R* is expressed as a ratio between different states, where A refers to the final state and B refers to the initial state:

10 $${\alpha }_{{\mathrm{A}}-{\mathrm{B}}}=\frac{{R}_{{\mathrm{A}}}}{{R}_{{\mathrm{B}}}}$$

The enrichment factor for the fractionation of phase A and B is commonly expressed in permil (‰), and can be calculated in relation to the fractionation factor *α*:

11 $${\varepsilon }_{{\mathrm{A}}-{\mathrm{B}}}\left({{\permil}}\right)=\left({\alpha }_{{\mathrm{A}}-{\mathrm{B}}}-1\right)\times 1,000$$

When *α*_A–B_ > 1 or *ε*_A–B_ > 0, phase A becomes enriched (heavier) during fractionation, whereas the phase A will become depleted (lighter) during fractionation if *α*_A–B_ < 1 or *ε*_A–B_ < 0.

The final isotopic composition of phase B (in ‰) after fractionation $({\delta }_{{{\mathrm{B}}}_{{{\mathrm{final}}}}})$ can be related to the initial composition of phase B $({\delta }_{{{\mathrm{B}}}_{{{\mathrm{initial}}}}})$, using the approximation of ref. ^60^:

12 $${\delta }_{{{\mathrm{B}}}_{{{\mathrm{final}}}}}={\delta }_{{{\mathrm{B}}}_{{{\mathrm{initial}}}}}+{\varepsilon }_{{\mathrm{A}}-{\mathrm{B}}}\mathrm{ln}\left(\,{f}_{{\mathrm{B}}}\right)$$

where ${f}_{{\mathrm{B}}}$ is the fraction of the material B that remains after fractionation. Once we calculate the fractionation factors due to degassing and carbonate precipitation^21^, we can then combine the fractionation factor from both reactions as follows:

13 $${\alpha }_{{\mathrm{A}}-{\mathrm{C}}}=\frac{{R}_{{\mathrm{A}}}}{{R}_{{\mathrm{C}}}}=\frac{{R}_{{\mathrm{A}}}}{{R}_{{\mathrm{B}}}}\times \frac{{R}_{{\mathrm{B}}}}{{R}_{{\mathrm{C}}}}={\alpha }_{{\mathrm{A}}-{\mathrm{B}}}\times {\alpha }_{{\mathrm{B}}-{\mathrm{C}}}$$

The enrichment factor for CO_2_ degassing is expressed as ${\varepsilon }_{{{{\mathrm{CO}}}}_{2}({\mathrm{g}})-{{{\mathrm{HCO}}}}_{3}}$, so the isotopic signature of HCO_3_ after reaction 1 $\left({\delta }_{{{{{\mathrm{HCO}}}}_{3}}_{{{\mathrm{Step}}}1}}\right)$ can be expressed as:

14 $${\delta }_{{{{{\mathrm{HCO}}}}_{3}}_{{{\mathrm{Step}}}1}}={\delta }_{{{{{\mathrm{HCO}}}}_{3}}_{{{\mathrm{initial}}}}}+\frac{{\varepsilon }_{{{{\mathrm{CO}}}}_{2}({\mathrm{g}})-{{{\mathrm{HCO}}}}_{3}}}{2}\mathrm{ln}\left(\,{f}_{{{{\mathrm{HCO}}}}_{3}}\right)$$

In equation (14), the enrichment factor is divided by two since we assume that half of the total enrichment occurs with reaction 1 and the other half occurs with reaction 2 (equation (15)). The enrichment factor for carbonate precipitation is expressed as ${\varepsilon }_{{{{\mathrm{CaCO}}}}_{3}-{{{\mathrm{HCO}}}}_{3}}$, so the isotopic signature of HCO_3_ after reaction 2 $\left({\delta }_{{{{{\mathrm{HCO}}}}_{3}}_{{{\mathrm{Step}}}2}}\right)$ is then:

15 $${\delta }_{{{{{\mathrm{HCO}}}}_{3}}_{{{\mathrm{Step}}}2}}={\delta }_{{{{{\mathrm{HCO}}}}_{3}}_{{{\mathrm{Step}}}1}}+\frac{{\varepsilon }_{{{{\mathrm{CaCO}}}}_{3}-{{{\mathrm{HCO}}}}_{3}}}{2}\mathrm{ln}\left(\,{f}_{{{{\mathrm{HCO}}}}_{3}}\right)$$

We then combine equations (14) and (15) to calculate the enrichment factor that encompasses the full set of reactions:

16 $${\delta }_{{{{{\mathrm{HCO}}}}_{3}}_{{{\mathrm{final}}}}}={\delta }_{{{{{\mathrm{HCO}}}}_{3}}_{{{\mathrm{initial}}}}}+{\varepsilon }_{{{\mathrm{C}}}_{{{\mathrm{loss}}}}-{{{\mathrm{HCO}}}}_{3}}\mathrm{ln}\left(\,{f}_{{{{\mathrm{HCO}}}}_{3}}\right)$$

where

17 $${\varepsilon }_{{{\mathrm{C}}}_{{{\mathrm{loss}}}}-{{{\mathrm{HCO}}}}_{3}}=\frac{{\varepsilon }_{{{{\mathrm{CO}}}}_{2}\left({\mathrm{g}}\right)-{{{\mathrm{HCO}}}}_{3}}+{\varepsilon }_{{{{\mathrm{CaCO}}}}_{3}-{{{\mathrm{HCO}}}}_{3}}}{2}$$

We calculate ${\alpha }_{{{{\mathrm{CaCO}}}}_{3}-{{{\mathrm{HCO}}}}_{3}}$ (equation (18)) and convert it to an enrichment factor, given the relationship between fractionation and enrichment factors in equation (11).

18 $${\alpha }_{{{{\mathrm{CaCO}}}}_{3}-{{{\mathrm{HCO}}}}_{3}}={\alpha }_{{{{\mathrm{CaCO}}}}_{3}-{{{\mathrm{CO}}}}_{2}({\mathrm{g}})}\times {\alpha }_{{{{\mathrm{CO}}}}_{2}({\mathrm{g}})-{{{\mathrm{HCO}}}}_{3}}$$

Enrichment factors are temperature dependent, so we calculated separate enrichment factors for the winter and summer samples using the seasonal range of temperatures from cold springs in the central Apennines^21,29^.

To estimate ${f}_{{\mathrm{B}}}$, we calculate the fraction of DIC lost due to CO_2_ degassing associated with secondary carbonate precipitation (*γ*_DIC_). Some CO_2_ degassing controlled by diffusion may also occur in the absence of carbonate precipitation^41^, although we have no way to estimate the magnitude of this process. However, previous studies suggest that the effect of diffusion-controlled degassing is negligible on isotope fractionation between gaseous CO_2_ (CO_2(g)_) and CO_2_ dissolved in water (CO_2(aq)_) because the reservoir of CO_2(aq)_ in solution is small between pH of 6 and 9, and the isotope fractionation between CO_2(g)_ and CO_2(aq)_ is small at 25 °C (ref. ^61^). By contrast, degassing caused by secondary carbonate precipitation produces a large fractionation between CO_2(g)_ and HCO_3_^−^ at ambient temperatures^62^.

Thus, we first calculate the amount of HCO_3_^−^ lost due to secondary carbonate precipitation (equations (3)−(9)). For each mol Ca^2+^ lost due to secondary carbonate precipitation, we lose 2 mol HCO_3_^−^ so that the concentration of HCO_3_^−^ before secondary precipitation can be expressed as:

19 $${\left[{{{\mathrm{HCO}}}}_{3}^{-}\right]}_{{{\mathrm{Initial}}}}=2\times \left({\left[{{{\mathrm{Ca}}}}^{2+}\right]}_{{{\mathrm{Initial}}}}-{\left[{{{\mathrm{Ca}}}}^{2+}\right]}_{{{\mathrm{Final}}}}\right)$$

where ${\left[{{{\mathrm{Ca}}}}^{2+}\right]}_{{{\mathrm{Final}}}}$ is the measured concentration of Ca in our water samples, and the initial concentration of Ca^2+^ before secondary carbonate precipitation $\left({\left[{{{\mathrm{Ca}}}}^{2+}\right]}_{{{\mathrm{Initial}}}}\right)$ is:

20 $${\left[{{{\mathrm{Ca}}}}^{2+}\right]}_{{{\mathrm{Initial}}}}=\frac{{\left[{{{\mathrm{Ca}}}}^{2+}\right]}_{{{\mathrm{Final}}}}}{{\gamma }_{{{\mathrm{CaC}}}{{\mathrm{O}}}_{3}}}$$

The initial DIC concentration ${\left[{{\mathrm{DIC}}}\right]}_{{{\mathrm{Initial}}}}$ can then be expressed as the sum of the measured DIC concentration ${\left[{{\mathrm{DIC}}}\right]}_{{{\mathrm{Final}}}}$ and ${\left[{{{\mathrm{HCO}}}}_{3}^{-}\right]}_{{{\mathrm{Initial}}}}$, so that the fraction of DIC lost due to secondary precipitation $\left({\gamma }_{{{\mathrm{DIC}}}}\right)$ can be expressed as:

$${\gamma }_{{{\mathrm{DIC}}}}=\frac{{\left[{{\mathrm{DIC}}}\right]}_{{{\mathrm{Final}}}}}{{\left[{{\mathrm{DIC}}}\right]}_{{{\mathrm{Initial}}}}}$$

Given the assumption that diffusion-controlled degassing should be negligible, we use ${\gamma }_{{{\mathrm{DIC}}}}$ as an approximation of ${f}_{{\mathrm{B}}}$.

### MEANDIR model

#### Scenario parameters

Scenario parameters for the inversion with MEANDIR are given in ref. ^21^ and details about the endmember compositions in Supplementary Text 2–4. Here we normalize all endmembers to the sum of measured concentrations (in μmol l^–1^) of riverine Ca^2+^, Mg^2+^, Na^+^, SO_4_^2−^ and DIC (equation (21)):

21 $${\chi }_{{\varSigma }^{+}}=2{\chi }_{{{{\mathrm{Ca}}}}^{2+}}+2{\chi }_{{{{\mathrm{Mg}}}}^{2+}}+{\chi }_{{{{\mathrm{Na}}}}^{+}}+2{\chi }_{{{{\mathrm{SO}}}}_{4}^{2-}}+{\chi }_{{{\mathrm{DIC}}}}$$

where $\chi$ designates the number of moles. Pyrite oxidation does not source any cations, although it is a source of SO_4_^2−^ and a sink of alkalinity, so we include SO_4_^2−^ in the normalization and treat the pyrite oxidation endmember independently of any weathering lithology^37^. Including DIC in the normalization also allows us to represent carbon endmembers that are decoupled from the weathering of lithologic endmembers.

Each variable is normalized by the sum of major dissolved cations given in equation (21) and is expressed in milliequivalents (mEq). To assure that the sum of variable ratios equals 1 and to maintain internal consistency for each endmember (equation (22)), we calculate the most abundant ratio through mass balance^37^.

22 $$1=\frac{2{\chi }_{{{{\mathrm{Ca}}}}^{2+}}}{\chi {\varSigma }^{+}}+\frac{2{\chi }_{{{{\mathrm{Mg}}}}^{2+}}}{\chi {\varSigma }^{+}}+\frac{{\chi }_{{{{\mathrm{Na}}}}^{+}}}{\chi {\varSigma }^{+}}+\frac{2{\chi }_{{{{\mathrm{SO}}}}_{4}^{2-}}}{\chi {\varSigma }^{+}}+\frac{{\chi }_{{{\mathrm{DIC}}}}}{\chi {\varSigma }^{+}}$$

For the DIC endmember, we assume DIC is sourced exclusively from biogenic carbon, metamorphic carbon, atmospheric carbon, cyclic inputs and carbonates. We calculate the DIC contribution for cyclic inputs and carbonate through charge balance and then convert the molar contributions to units of mEq. For the biogenic carbon endmember, we select a broad range of isotopic signatures for C3 plants that are dominant in central Italy^63^ and account for the enrichment of δ^13^C due to plant degradation. DO^13^C estimates for the Tyrrhenian side of the Apennines are not available, although the importance of C4 crops such as maize within the Tevere catchment^64^ would suggest that waters may have more enriched isotopic signatures relative to those of C3 plants.

#### Inversion approach

We invert all 100 samples for which we have all the required elemental and isotopic measurements. To select successful runs, we employ two separate criteria. First, we select from 1 × 10^6^ simulations those with reconstructed chemical compositions that fall within prescribed misfit ranges (the ‘iterate over samples’ approach in MEANDIR). The final value and uncertainty are then calculated as the median and interquartile range of all accepted simulations. For all ion data, we allow reconstructed value within 75–125% of the observations^21^. For δ^34^S and δ^13^C isotopes, we allow a maximum misfit of ±2‰ and a misfit smaller than or equal to 0.05 for F^14^C (ref. ^21^). Given these bounds, 41% of winter samples and 57% of summer samples produce successful simulations (Supplementary Text 5). Other samples have misfits that are greater than the acceptable limits (Supplementary Text 5 and Supplementary Fig. 3)^21^. Second, we follow previous approaches of inversion models^38,65^ and select the best 5% of simulations to calculate the inversion result from 1 × 10^4^ simulations. This approach produces successful inversions for all samples. From all 5% of accepted simulations, we calculate minimum and maximum misfit values^21^.

In all figures we present data from the inversions that fit within the prescribed misfit bounds (those samples with successes under the iterate-over-samples approach). These results represent the most conservative treatment of the inversion because it requires successful iterations to reproduce the observations within some misfit bounds. In the supplement, we provide the MEANDIR model fractional output using the selection of the top 5% of simulations^21^ and an alternative to summary Fig. 4 (Extended Data Fig. 6) that includes the full sample set (samples with successes using the iterate-over-samples approach and the results from the iterate-over-endmembers approach for the remaining samples).

#### Model outputs and calculations

The fractional contribution from each endmember is expressed as the median value from all accepted simulations, which we convert to concentrations, to compare results from all sampling locations and different spatial scales. To estimate inorganic CO_2_ consumption and production, we calculate the concentration of CO_2_ (in μmol l^–1^) that is sequestered or released from weathering. For silicate weathering, the consumed CO_2_ concentration ${\left[{{{\mathrm{CO}}}}_{2}\right]}_{{{\mathrm{Sil}}}}$ is calculated by assuming that each charge-equivalent ion sequesters 0.5 mol CO_2_ (refs. ^38,65^), such that

23 $${\left[{{{\mathrm{CO}}}}_{2}\right]}_{{{\mathrm{Sil}}}}=-0.5\left(2[{{{\mathrm{Ca}}}}^{2+}]+2[{{{\mathrm{Mg}}}}^{2+}]+[{{{\mathrm{Na}}}}^{+}]\right)$$

For metamorphic CO_2_ release ${\left[{{{\mathrm{CO}}}}_{2}\right]}_{{{\mathrm{Meta}}}}$, every charge equivalent of metamorphic DIC $\left({\left[{{\mathrm{DIC}}}\right]}_{{{\mathrm{Meta}}}}\right)$ acts as a source for one mol CO_2_ to the atmosphere.

24 $${\left[{{{\mathrm{CO}}}}_{2}\right]}_{{{\mathrm{Meta}}}}={\left[{{\mathrm{DIC}}}\right]}_{{{\mathrm{Meta}}}}$$

For pyrite oxidation ${\left[{{{\mathrm{CO}}}}_{2}\right]}_{{{\mathrm{Pyr}}}}$, we consider each charge equivalent of SO_4_^2−^ as a source of 0.5 mol CO_2_ because of the alkalinity consumption by sulfuric acid.

25 $${\left[{{{\mathrm{CO}}}}_{2}\right]}_{{{\mathrm{Pyr}}}}=0.5\left(2\left[{\mathrm{S}}{{\mathrm{O}}}_{4}^{2-}\right]\right)$$

We do not include K^+^ in our unmixing model or calculations because its inclusion reduces the number of successful runs in the MEANDIR model by more than 50% without substantially affecting the estimates of weathering and carbon sources.

Where discharge measurements were available from the regional hydrologic authorities^21^, we divided these measurements by the upstream drainage area to calculate run-off and converted the CO_2_ concentrations into fluxes, expressed in tons of carbon (tC km^–2^ yr^–1^) (ref. ^21^). Daily discharge estimates were averaged over the two summer (July–August) or winter (March–April) months during which we sampled.

## Online content

Any methods, additional references, Nature Portfolio reporting summaries, source data, extended data, supplementary information, acknowledgements, peer review information; details of author contributions and competing interests; and statements of data and code availability are available at 10.1038/s41561-024-01396-3.

### Supplementary information

Supplementary Information Supplementary Fig. 1 and Text.

## Data Availability

The datasets used in this paper are available from 10.5880/GFZ.4.6.2024.001 (ref. ^21^) and 10.13127/class.1.0 (earthquake data)^49^.

## Code availability

The MEANDIR model and user guide^3^ are available at https://github.com/PrestonCosslettKemeny/MEANDIR.
