# Supplementary material for: Deep CO2 release and the carbon budget of the central Apennines modulated by geodynamics
Source: Nat Geosci. 2024 Apr 19;17(5):465–71. doi: 10.1038/s41561-024-01396-3 (PMC11093751; doi:10.1038/s41561-024-01396-3)
Supplement: Supplementary file 1 — Supplementary Fig. 1 and Text. [file 41561_2024_1396_MOESM1_ESM.pdf]

nature geoscience

Article https://doi.org/10.1038/s41561-024-01396-3

Deep CO_2 release and the carbon budget of the central Apennines modulated by geodynamics

In the format provided by the authors and unedited

This file includes:

Supplementary Text

S1: Geologic Setting

S2: Isotopic Signatures of Sulfate

S3: Isotopic Endmember Constraints

S4***:*** Elemental Endmember Constraints

S5: Model Misfits

S6: Supplementary Figures

S1: Geologic Setting

The surface geology of the Central Apennines is dominated by a Mesozoic sedimentary sequence[1,2], covered by Cenozoic syn-orogenic deposits. Late Triassic evaporites (halite, gypsum, anhydrite) form the base of the Mesozoic passive margin sequence (Burano Formation) and cover less than 1% of the surface in Italy[3] (Figure 1e). The Burano Formation is overlain by Triassic to Jurassic carbonate platform sediments (Calcare Massiccio Formation), Upper Cretaceous pelagic chert limestones (Maiolica Formation)[4], and Miocene carbonates[3]. The stack of syn-orogenic sediments is comprised of siliciclastic-rich turbidite sequences of the Modino-Cervarola Unit and Laga Formation[5], overlain by allochthonous remnants of the Cretaceous Ligurian Tethys Ocean (Ligurian Unit)[6]. The youngest deposits exposed in the study area are Pliocene and Quaternary volcanics on the western margin of the peninsula[7], and Plio-Pleistocene lacustrine and alluvial deposits[3]. The Tevere and Aterno-Pescara catchments cover this range of lithologies and span across the geodynamic gradient (Figure 1).

S2: Isotopic Signatures of Sulfate

We illustrate SO_4 isotopic signatures from the studied catchments, relative to two endmembers that reflect either pyrite oxidation or evaporite weathering. Both the Aterno-Pescara and Tevere Rivers illustrate similar ranges of $\delta^{34}S$ and $\delta^{18}O(SO_4)$ (Figure E2). Carbonate and mixed samples cover the full range of measured values, whereas the Tevere siliciclastic samples consistently illustrate some of the most depleted signatures. The majority of samples lie between the evaporite and pyrite oxidation endmembers, suggesting that sulfate in the river waters is generally a mixture of the two endmembers. The majority of groundwater samples, and overall 35% of the winter samples and 30% of the summer samples lie within or close to the evaporite endmember field, even though evaporites are not exposed at the surface within the field area (Figure 1e). This observation demonstrates that river water from tributaries in both catchments and some Tevere springs with evaporite $\delta^{34}S$ and $\delta^{18}O(SO_4)$ signatures interact with rising fluids enriched in H_2S (ref.[8]).

S3: Isotopic Endmember Constraints

We constrain the isotopic composition of $\delta^{34}S$ for the pyrite oxidation endmember from Apenninic pyrite samples[9]. To constrain the evaporite endmember, we used regional estimates of $\delta^{34}S$ (refs. [10,11]) for Triassic evaporites (Table S4).

Sulfidic caves hosted in the central Apennines can contain a variety of sulfur-bearing compounds that reflect abiotic and microbial recycling between sulfide (H_2S) and sulfate (H_2SO4 or $CaSO_4$) (ref. [12]). Such microbial processes can produce large fractionations between sulfur-bearing compounds[13,14]. $\delta^{34}S$ measurements exist for secondary gypsum in Italian sulfidic caves[8]; however, we do not have evidence that this process is volumetrically relevant for the

central Apennines rivers, and therefore exclude these sources of sulfate from our isotopic endmembers.

S4: Elemental Endmember Constraints

We prescribed four lithologic endmembers in the inversion model: carbonates, silicates, evaporites, and pyrite (Table S4). We include a pyrite endmember to distinguish between sources of sulfate from evaporites and oxidative weathering of sulfides. In addition to the lithologic endmembers, we also include cyclic inputs to account for dissolved ions from meteoric water. Finally, we include three carbon endmembers that allow us to represent the exchange of CO_2 between the atmosphere and the river: 1) biogenic carbon, which we broadly define as carbon sourced from carbonic acid or biologic activity in the soil or river, 2) carbon from metamorphic reactions in the subsurface, and 3) atmospheric carbon.

Inversion endmembers (Table S4) were constrained from local bedrock[10,11,15–17], local meteoric water[18,19], carbon sources[20–22], or were based on global endmember compositions[23] if local constraints were unavailable or did not reflect endmember compositions for the entire field area. Siliciclastic-rich turbidite deposits in the Apennines can contain a substantial carbonate fraction[15], and bulk chemistry of carbonates was not available for the Central Apennines, so we used generic silicate and carbonate endmembers[24] that were also calculated with the same normalization variables (see Table 13, scenario KH-2).

Because this study is focused on understanding how the exported load of riverine dissolved ions contributes to the carbon budget in the Central Apennines, we incorporated only endmembers that contribute ions or elements to the riverine dissolved load. The widespread presence of travertine deposits in the Central Apennines[25] suggests that secondary precipitation of calcite is an important process in the Central Apennines. However, we are unable to chemically or isotopically differentiate between primary and secondary carbonate within the inversion model.

S5. Model Misfits

The analysis of misfits from the 5% best-fitting simulations (using the "iterate over endmember" option in MEANDIR) show that our model reproduces well all of the major ion data with misfits of < 20%. In contrast, misfits for the ion data tended to be larger. Two main groups of samples produced misfits that were larger than the desired cutoff (see method).

1) *Samples with a mismatch of $F^{14}C$ and/or $\delta^{34}S$ signatures*.

From the 100 total samples run in MEANDIR, 39 samples (39%) fail because the $F^{14}C$ values cannot be reproduced with reconstructed values being lower than observed values (negative

misfits). All but one of these samples invariably have $F^{14}C > 0.75$ and at the same time relatively depleted $\delta^{13}C$ of < -8.0 (Figure S1). Carbonate weathering with carbonic acid should produce dissolved inorganic carbon (DIC) of 0.5, which represents a mixture between "dead" radiocarbon from the carbonate and modern carbon from the acid. Within our set of endmembers, $F^{14}C$ higher than 0.5 can only be achieved by silicate weathering. However, Ca/Na ratios are high (average Ca/Na= 6.5), and we do not have evidence for a substantial contribution of non-carbonate Ca-sources, such as from gypsum. As a result, the inversion cannot find results that both predict a high carbonate weathering and very modern $F^{14}C$ for most of our samples – which explains the high number of failed runs. We hypothesize that these modern DIC signatures could reflect multiple cycles of dissolution and precipitation, which represents an additional process that the model does not account for.

Of the 39 samples with high $F^{14}C$ misfits, 10 samples also have high misfit between the observed and reconstructed $\delta^{34}S$ values, with reconstructed values being higher than the observed values (positive misfits). All samples with these high misfits have observed depleted or moderately enriched $\delta^{34}S$ values ($\delta^{34}S < 6.5$). Two samples (25 and 35) have high misfits only for $\delta^{34}S$, but they also have high $F^{14}C$ values ($F^{14}C > 0.65$). As explained above, high $F^{14}C$ are consistent with low carbonate weathering. Thus, with a high Ca/Na ratio, Ca and SO_4 may be modeled to derive from gypsum. However, high gypsum contributions would imply very enriched $\delta^{34}S$ values (the gypsum endmember has $\delta^{34}S$ = 10.6 – 17.4‰). Thus, balancing observations of very modern $F^{14}C$ values, high Ca/Na ratios, and depleted or only moderately enriched $\delta^{34}S$ can lead to misfits in $F^{14}C$ and/or $\delta^{34}S$. Decreasing $\delta^{34}S$ in the gypsum endmember may improve the model misfits, but we lack local constraints on the isotopic composition of gypsum to justify this approach. Moreover, we do not know whether the reason for the modern $F^{14}C$ value could be the re-precipitation and re-dissolution of carbonates.

2) *Samples with a mismatch of $\delta^{13}C$ signatures*

These six samples are typically from locations that either have depleted $\delta^{13}C$ values ($\delta^{13}C$ = -7.9 – -15.3‰) and positive misfits or springs with enriched $\delta^{13}C$ values ($\delta^{13}C$ = 3.0 – 10.8‰) and negative misfits. With two exceptions, these samples all have old carbon signatures ($F^{14}C$ <0.37). To achieve these "old" carbon signatures, the model requires a high metamorphic carbon input with $\delta^{13}C$ = -5 – 2‰, which cannot be fit well with either the more depleted or more enriched observations. It is possible that the isotopic composition of our metamorphic endmember does not fit across the study area, but we have no data for more local isotopic variations that would deviate from this expectation[22]. Because the majority of our samples with low $F^{14}C$ are well fit, we prefer to use an endmember range that is supported by local data rather than widening the range until they fit all of our datapoints.

S6: Supplementary Figures

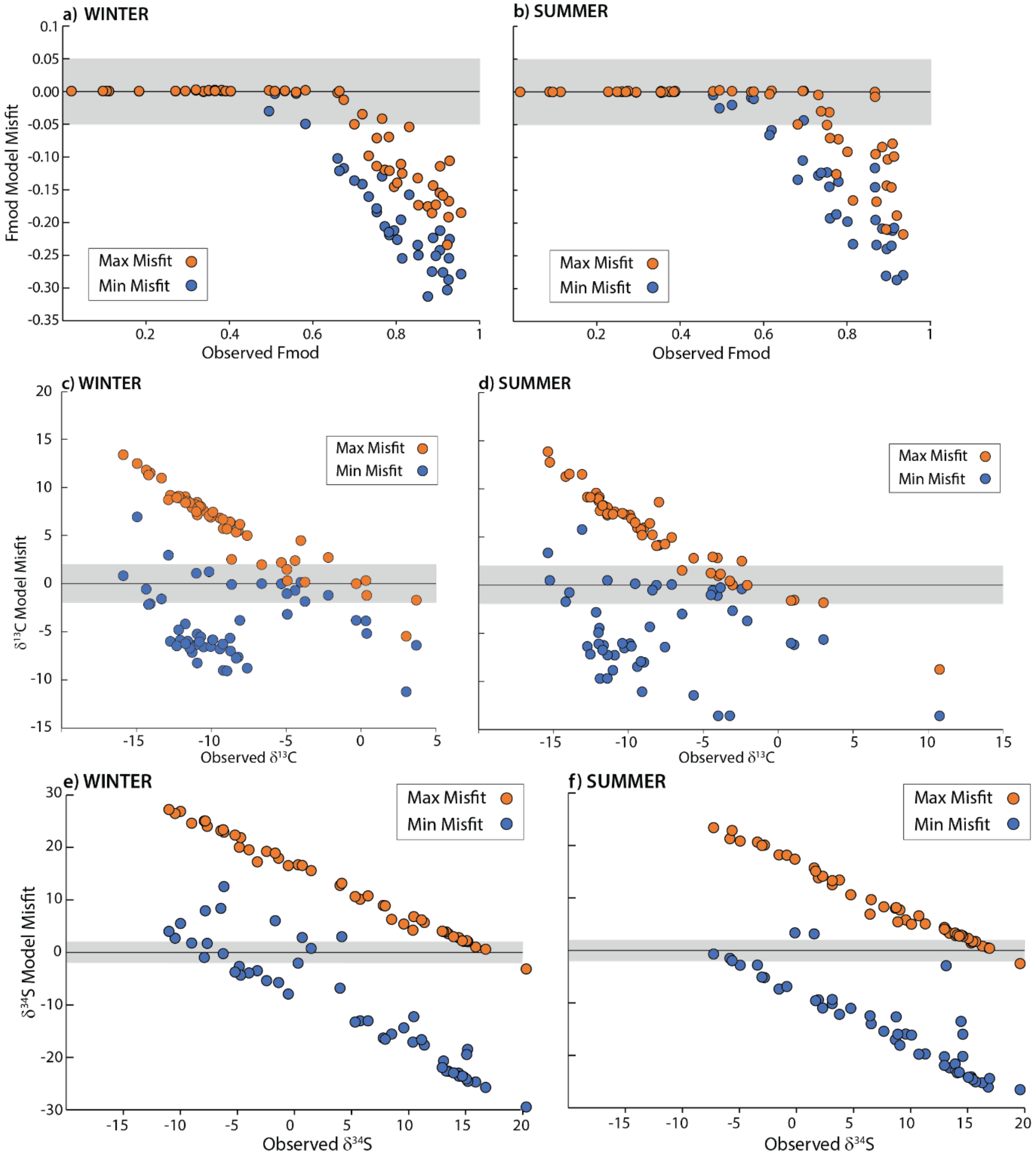

Figure S1. MEANDIR model misfits for samples run using the "iterate over endmembers" approach (Table S15). The misfit reflects the difference between the observed and reconstructed isotopic values for $F^{14}C$ a) winter and b) summer samples, for $\delta^{13}C$ c) winter and summer samples, and for $\delta^{34}S$ e) winter and f) summer samples.

References

1. Bigi, G. *et al.* Structural model of Ital, sheet no. 3, 1:500,000. (1992).
2. Bigi, G. *et al.* Structural Model of Italy, sheet 4, scale 1: 500,000. *CNR Quaderni de ''La Ricerca Scientifica* vol. 114 (1992).
3. Cosentino, D., Cipollari, P., Marsili, P. & Scrocca, D. Geology of the central Apennines: a regional review. *J. virtual Explor.* **36**, 1–37 (2010).
4. Minissale, A., Vaselli, O., Tassi, F., Magro, G. & Grechi, G. P. Fluid mixing in carbonate aquifers near Rapolano (central Italy): Chemical and isotopic constraints. *Appl. Geochemistry* **17**, 1329–1342 (2002).
5. Ricci Lucchi, F. Miocene paleogeography and basin analysis in the Periadriatic Apennines: Petroleum Exploration Society of Libya. (1975).
6. Pini, G. A. Tectonosomes and Olistostromes in the Argile Scagliose of the Northern Apennines. *Geol. Soc. Am. Spec. Pap.* **335**, 70 (1999).
7. Cavinato, G. P., Cosentino, D., De Rita, D., Funiciello, R. & Parotto, M. Tectonic-sedimentary evolution of intrapenninic basins and correlation with the volcano-tectonic activity in Central Italy. *Mem. Descr. della Cart. Geol. d'Italia* **49**, 63–76 (1994).
8. D'Angeli, I. M. *et al.* Sulfuric acid caves of Italy: A review. *Geomorphology* **333**, 105–122 (2019).
9. Schwarcz, H. P. & Cortecci, G. Isotopic analyses of spring and stream water sulfate from the Italian Alps and Apennines. *Chem. Geol.* **13**, 285–294 (1974).
10. Boschetti, T., Venturelli, G., Toscani, L., Barbieri, M. & Mucchino, C. The Bagni di Lucca thermal waters (Tuscany, Italy): An example of Ca-SO4 waters with high Na/Cl and low Ca/SO4 ratios. *J. Hydrol.* **307**, 270–293 (2005).
11. Boschetti, T., Cortecci, G., Toscani, L. & Iacumin, P. Sulfur and oxygen isotope compositions of Upper Triassic sulfates from northern Apennines (Italy): Paleogeographic and hydrogeochemical implications. *Geol. Acta* **9**, 129–147 (2011).
12. Galdenzi, S. & Maruoka, T. Gypsum deposits in the Frasassi Caves, Central Italy. *J. Cave Karst Stud.* **65**, 111–125 (2003).
13. Canfield, D. E. Isotope fractionation by natural populations of sulfate-reducing bacteria. *Geochim. Cosmochim. Acta* **65**, 1117–1124 (2001).
14. Zerkle, A. L., Jones, D. S., Farquhar, J. & Macalady, J. L. Sulfur isotope values in the sulfidic Frasassi cave system, central Italy: A case study of a chemolithotrophic S-based ecosystem. *Geochim. Cosmochim. Acta* **173**, 373–386 (2016).
15. Dinelli, E., Ł, F. L., Mordenti, A. & Paganelli, L. Geochemistry of Oligocene – Miocene sandstones of the northern Apennines (Italy) and evolution of chemical features in relation to provenance changes. **127**, 193–207 (1999).
16. Zuppi, G. M. & Bortolami, G. C. Hydrogeology: a privileged field for environmental stable isotopes applications. Some Italian examples. *Rend. Soc. Ital. Miner. Pet.* **38**, 1197–1212 (1982).
17. Cavarretta, G. & Lombardi, G. Origin of sulphur in minerals and fluids from Latium (Italy): isotopic constraints. *Eur. J. Mineral.* **4**, 1311–1330 (1992).
18. Vet, R. *et al.* A global assessment of precipitation chemistry and deposition of sulfur, nitrogen, sea salt, base cations, organic acids, acidity and pH, and phosphorus. *Atmos. Environ.* **93**, 3–100 (2014).

19. Cortecci, G. & Longinelli, A. Isotopic composition of sulfate in rain water, Pisa, Italy. *Earth Planet. Sci. Lett.* **8**, 36–40 (1970).
20. Chiodini, G., Frondini, F., Cardellini, C., Parello, F. & Peruzzi, L. Rate of diffuse carbon dioxide Earth degassing estimated from carbon balance of regional aquifers: The case of central Apennine, Italy. *J. Geophys. Res. Solid Earth* **105**, 8423–8434 (2000).
21. Tesi, T., Langone, L., Giani, M., Ravaioli, M. & Miserocchi, S. Source, diagenesis, and fluxes of particulate organic carbon along the western adriatic Sea (mediterranean Sea). *Mar. Geol.* **337**, 156–170 (2013).
22. Chiodini, G. *et al.* Carbon dioxide Earth degassing and seismogenesis in central and southern Italy. *Geophys. Res. Lett.* **31**, (2004).
23. Burke, A. *et al.* Sulfur isotopes in rivers: Insights into global weathering budgets, pyrite oxidation, and the modern sulfur cycle. *Earth Planet. Sci. Lett.* **496**, 168–177 (2018).
24. Kemeny, P. C. & Torres, M. A. Presentation and applications of mixing elements and dissolved isotopes in rivers (MEANDIR), a customizable MATLAB model for Monte Carlo inversion of dissolved river chemistry. *Am. J. Sci.* **321**, 579–642 (2021).
25. Minissale, A. *et al.* Geochemistry of Quaternary travertines in the region north of Rome (Italy): structural, hydrologic and paleoclimatic implications. *Earth Planet. Sci. Lett.* **203**, 709–728 (2002).
